# *Campylobacter* in backyard poultry: hidden risks and public health implications

**DOI:** 10.2478/jvetres-2026-0016

**Published:** 2026-03-30

**Authors:** Aleksandra Kobuszewska, Beata Izabela Wysok, Paulina Przyborowska

**Affiliations:** Department of Veterinary Public Health, University of Warmia and Mazury, 10-917 Olsztyn, Poland

**Keywords:** antimicrobial resistance, campylobacteriosis, *Campylobacter*, genetic diversity, smallholder poultry

## Abstract

**Introduction:**

Although risk factors associated with *Campylobacter* infection in poultry have been recognised, a notable gap exists associated with this infection in backyard poultry.

**Material and Methods:**

Birds were examined by taking 315 cloacal swabs in six small, backyard poultry flocks of ducks, geese and ornamental chickens to determine the occurrence, genetic diversity and antimicrobial susceptibility of *Campylobacter* spp. Genomic diversity was assessed by sequencing the short variable region of the *flaA* gene and by virulence gene profiling. Antimicrobial susceptibility was analysed by the minimal inhibitory concentration method with the agar dilution technique.

**Results:**

The prevalence of *Campylobacter* was 5.1%, with positive findings limited to ducks and ornamental chickens. A total of 16 isolates were recovered, comprising 14 of *C. jejuni* and 2 of *C. coli*. Duck isolates showed higher genetic diversity (Simpson’s diversity index (SDI) = 1.000), but ornamental chicken isolates were also not homogeneous (SDI = 0.911). The most frequently detected virulence determinants were *flaA* (100.0%), *ciaB* (75.0%), *cadF* (62.5%) and *cdtC* (62.5%). Antimicrobial resistance was most frequently to ciprofloxacin (93.8%), and to tetracycline (43.8%) and erythromycin (43.8%) in many instances, while multidrug resistance was found in 18.8% of isolates.

**Conclusion:**

The results will contribute to a more comprehensive understanding of the ecology and transmission dynamics of *Campylobacter* under the One Health framework.

## Introduction

*Campylobacter* is a genus of Gram-negative, microaerophilic bacteria that currently encompasses 27 recognised species (36). Members of the genus are widely distributed and are commonly isolated from diverse sources, including human and animal populations, as well as a wide array of food products, especially those of animal origin. *Campylobacter jejuni* and *C. coli* are recognised as historically significant pathogens, particularly as zoonotic pathogens that can cause campylobacteriosis in both humans and animals (29).

*Campylobacter* species warrant particular attention as major foodborne pathogens and causative agents of campylobacteriosis, a bacterial gastroenteritis imposing a substantial public health burden in both industrialised and developing countries (58). Food-producing animals are a primary source of *Campylobacter* transmitted to humans primarily through consumption (43). Cattle, sheep and pigs are also reservoirs of these bacteria. Despite the environmental ubiquity of these pathogens, the primary route of human infection is through foodborne transmission, where raw or inadequately processed foods constitute the most significant sources of exposure (20).

In humans, campylobacteriosis typically presents as an acute gastrointestinal illness characterised by diarrhoea, abdominal pain, fever and malaise. While most cases are self-limiting, there can be severe outcomes, particularly in young children, the elderly or immunocompromised individuals (38). In rare instances, *C. jejuni* infection can lead to post-infectious complications such as Guillain-Barré syndrome (GBS), a serious autoimmune neuropathy that may result in long-term neurological impairment or paralysis (57).

Public health concerns are compounded by a reported rise in campylobacteriosis incidence across Europe. According to the European Food Safety Authority (EFSA) report of 2023, campylobacteriosis was confirmed as the most prevalent zoonosis in EU countries, being so for the 19^th^ consecutive time. It accounted for 58.9% of all human cases of zoonoses that were reported and confirmed in the EU in 2023. Alarmingly, the EFSA report also revealed that the overall trend in human *Campylobacter* infections had not changed significantly and had remained stable in 2019–2023. Moreover, in 2023, *Campylobacter* infections were associated with a high rate of hospitalisation (23.9%) and a case-fatality rate of 0.05% (13).

Poultry, particularly chickens, act as the primary reservoir of *Campylobacter* bacteria which commonly colonise their intestines, often without causing overt clinical symptoms (22). Contamination of edible tissue frequently occurs during slaughter and processing, leading to the presence of *Campylobacter* in raw poultry products (53). In recent years, research has predominantly focused on large-scale poultry farming as a critical source of *Campylobacter* infections, driven by its central role in the global food supply chain and the intensive rearing conditions it sets (21). The significance of *Campylobacter* to humans is reflected in current legislation, which mandates its monitoring throughout the food chain, from primary production (farm and aquaculture animals), through harvesting/slaughter, to processing and distribution. The EU has implemented a process hygiene criterion (PHC) for *Campylobacter*, and a regulatory limit of 1,000 CFU/g on the neck skin of chilled broiler carcasses was established by Commission Regulation (EC) No. 2073/2005. Broiler carcass contamination in the EU has been estimated at 40.0% (13). The results of *Campylobacter* PHC monitoring indicate that poultry is a significant source of campylobacteriosis in the EU (13). Despite the recognition of this risk in poultry, a notable gap exists in the literature: the risk factors associated with *Campylobacter* contamination of the meat of backyard poultry have not been specifically addressed to date.

It has been argued that intensive farming practices increase the risk of zoonoses and their spread because of easier transmission between animals. However, industrial farms are subject to rigorous biosecurity measures, including controlled access, regular cleaning and disinfection protocols, which help mitigate the risk of zoonotic transmission (55). Poultry breeding has been intensified to meet the growing demand for meat and eggs, yet at the same time, these intensive systems have attracted significant criticism from those creating the demand, creating strong pressure in several countries to transform animal production into a more extensive model, such as backyard farming (49). Although small-scale poultry production is usually less efficient in terms of feed conversion compared to industrial systems, the growing interest in backyard farming reflects a concern for animal welfare, food quality, and reduced use of chemicals, which are also important dimensions of sustainable agriculture.

Backyard poultry are often kept in close proximity to other domestic animals, such as dogs and cats, and frequently lack structural barriers preventing contact with wildlife, including rodents and wild birds. These animals may act as mechanical vectors of *Campylobacter* spp. and other zoonotic agents (44, 53). Such limitations in biosecurity may increase the likelihood of pathogen introduction from external sources and facilitate transmission within backyard flocks. Moreover, frequent human–poultry interactions, combined with less stringent hygiene practices compared to commercial production systems, may further contribute to the risk of transmission to humans within the household (21).

In addition to not preventing environmental exposure, the open structure of small-scale poultry rearing pens renders effective disinfection exceedingly difficult, and hygiene protocols are frequently inadequate. The “all-in, all-out” management strategy is a critical component of biosecurity plans in commercial operations. This strategy involves complete flock removal, followed by thorough cleaning, disinfection and a fallow period before the introduction of a new cohort. This approach is designed to interrupt disease transmission cycles by allowing pathogens to die off in the absence of hosts. However, implementing such practices in backyard or smallholder systems is rarely practical, as birds are often added incrementally rather than a flock being replaced entirely. This continuous turnover prevents full sanitation and contributes to the persistence of infectious agents in the environment (54). These limitations are further exacerbated by a general lack of awareness regarding zoonotic risks among backyard flock owners (19). Studies indicate that backyard poultry environments often have higher microbial loads than industrial systems, partly due to the absence of targeted interventions such as vaccination, prophylactic treatments or water decontamination strategies (16). Moreover, the growing popularity of backyard poultry rearing as a sustainable or recreational activity, particularly in urban and peri-urban areas, ascribes it an epidemiological role in the context of campylobacteriosis (29). While large poultry operations are the most studied reservoirs of *Campylobacter* spp., small-scale rearing represents a critical yet underexplored link in the zoonotic transmission chain (8). Its contribution to transmission is primarily due to a lack of biosecurity and its birds’ frequent interactions with other domestic and wild animals (23).

Whether human campylobacteriosis is zoonotic in origin – as it most frequently is – or is not, standard treatment protocols typically involve macrolide antibiotics, where erythromycin is the first-line agent, and azithromycin is used as an alternative (8). Fluoroquinolones, such as ciprofloxacin, are also frequently employed in the empirical treatment of undiagnosed cases of bacterial diarrhoea. In certain instances, tetracyclines and phenicols are alternative therapeutic options. Systemic campylobacteriosis is generally treated with aminoglycosides or carbapenems. Although third-generation cephalosporins are sometimes used as alternatives to fluoroquinolones, they are not effective in treating *Campylobacter* spp. bacteraemia, except in cases involving *C. fetus* (15). Increasing antimicrobial resistance (AMR), particularly among *C. jejuni* and *C. coli* strains, has become a major concern (4, 15). Resistant isolates are increasingly detected in food, the environment and human infections, with high resistance rates especially to ciprofloxacin and tetracycline (15). Although resistance to chloramphenicol and gentamicin remains relatively low, the geographical variability of AMR patterns, driven by local antimicrobial use, highlights the importance of a One Health approach to address this issue.

Molecular tools are central to the One Health surveillance of *Campylobacter* AMR and transmission, enabling source attribution across human, animal and environmental compartments. Among these, molecular characterisation through the sequencing of the short variable region of the *flaA* (flagellin A) gene (*flaA*-SVR) is widely used in molecular epidemiology for fine-scale strain differentiation, and was therefore applied to assess the genetic diversity among *Campylobacter* isolates. This method is widely used in molecular epidemiology because it enhances the understanding of transmission dynamics in environments characterised by complex host and ecological interactions (10). Previous studies have shown that *Campylobacter* spp., particularly *C. jejuni* and *C. coli*, display considerable genetic diversity, which complicates outbreak investigations and source attribution. The *flaA*-SVR sequencing approach is widely used in molecular epidemiology for the differentiation of *Campylobacter* strains. Most existing data originate from commercial poultry or clinical settings, leaving a gap in our understanding of the *Campylobacter* population structure in backyard poultry flocks. The present study addresses this gap by applying *flaA*-SVR sequencing to isolates obtained from non-commercial flocks, providing novel data on strain diversity and potential reservoirs outside intensive farming systems (25). The aim of this study was to fill the knowledge gap regarding the prevalence and genetic characteristics of *Campylobacter* spp. in backyard poultry by evaluating the potential role of birds as reservoirs for zoonotic transmission. By characterising the prevalence, antimicrobial resistance, and genetic diversity of isolates, this research provides critical insights into the epidemiological significance of small avian populations.

## Material and Methods

### Sampling

A total of 315 cloacal swab samples were collected from birds reared in six small backyard holdings in Poland. The sampling sites were selected based on their accessibility, the owners’ willingness to participate in the study, and collective fulfilment of a wide range of conditions representative of small-scale production, including different species composition, management practices and geographic settings (rural and semi-urban). The sampling was conducted in the north-eastern region of Poland, and all samples were collected during the spring season. Samples were obtained from ducks, geese and ornamental chickens of multiple breeds. The latter were kept primarily for aesthetic or hobby purposes rather than for food production. Cloacal swabbing was performed as part of standard bird handling procedures to minimise stress. Some of the rearing locations housed other animal species alongside poultry, while others maintained bird-only environments. Cloacal swabs were placed in Amies transport medium with charcoal (Deltalab, Barcelona, Spain) and transported to the laboratory for subsequent analysis.

### Isolation of bacteria

Bacterial strains were isolated following the protocol outlined in EN ISO 10272-1:2017 (24), which describes a horizontal method for detecting *Campylobacter* spp. The swabs were transferred to 9 mL of Bolton broth (Oxoid, Basingstoke, UK). Enrichment cultures were incubated at 37°C for 4 h and at 41.5°C for 44 ± 4 h under microaerobic conditions (5% O_2_, 10% CO_2_, 85% N). A loopful of the enriched suspension was plated onto modified charcoal cefoperazone deoxycholate agar and Karmali agar (both from Oxoid). The plates were incubated for 24–48 h under the same microaerobic conditions. Colonies were selected for further analysis based on morphological features characteristic of *Campylobacter*, namely small, round or irregularly shaped, grey-toned, moist colonies with a glistening or spreading appearance (14). Single colonies were collected and subjected to confirmatory tests, including a microscopic examination and analyses of oxidase activity, motility and absence of growth under strictly aerobic conditions at 25°C. The isolates were subcultured only once to minimise potential genetic alterations during repeated passages and were subsequently stored at –80°C in defibrinated horse blood (Oxoid) supplemented with glycerol (80:20 v/v).

### Identification

*Campylobacter* isolates were identified to species level using a PCR-based approach. The isolates were cultured on Columbia agar (Oxoid) supplemented with blood, and a single colony was suspended in 1 mL of sterile water and centrifuged at 13,000 × *g* for 1 min. The resulting pellet was resuspended in Tris buffer (Merck, Darmstadt, Germany), and genomic DNA was extracted using the Genomic Mini Kit (A&A Biotechnology, Gdańsk, Poland) according to the manufacturer’s protocol. The concentration and purity of the DNA were assessed spectrophotometrically, and the extracted DNA was used as a template for species-specific PCR assays. The primers used in these assays are listed in Table 1.

**Table 1.** List of primers used in the study and thermocycler settings for the amplification of *Campylobacter* virulence genes

Target gene	Sequences (5ʹ–3ʹ)	Annealing temperature (°C)	Reference
16S rRNA for *Campylobacter* spp.	F-ATCTAATGGCTTAACCATTAAAC R-GGACGGTAACTAGTTTAGTATT	55	(56)
*mapA* for *C. jejuni*	F-CTATTTTATTTTTGAGTGCTTGTG R-GCTTTATTTGCCATTTGTTTTATTA	55	(56)
*ceuE* for *C. coli*	F-AATTGAAAATTGCTCCAACTATG R-TGATTTTATTATTTGTAGCAGCG	55	(56)
*flaA*	F-AATAAAAATGCTGATAAAACAGGTG R-TACCGAACCAATGTCTGCTCTGATT	53	(9)
*flaA-SVR*	F-CTATGGATGAGCAATT(AT)AAAAT R-CAAG(AT)CCTGTTCC(AT)ACTGAAG	50	(39)
*cadF*	F-TTGAAGGTAATTTAGATATG R-CTAATACCTAAAGTTGAAAC	45	(30)
*iam*	F-GCGCAAAATATTATCACCC R-TTCACGACTACTATGCGG	50	(9)
*ciaB*	F-TGCGAGATTTTTCGAGAATG R-TGCCCGCCTTAGAACTTACA	62	(5)
*pldA*	F-AAGCTTATGCGTTTTT R-TATAAGGCTTTCTCCA	47	(9)
*cdtA*	F-CCTTGTGATGCAAGCAATC R-ACACTCCATTTGCTTTCTG	55	(9)
*cdtB*	F-CAGAAAGCAAATGGAGTGTT R-AGCTAAAAGCGGTGGAGTAT	55	(9)
*cdtC*	F-CGATGAGTTAAAACAAAAAGATA R-TTGGCATTATAGAAAATACAGTT	53	(9)
*pebA*	F-GCTCTAGGTGCTTGTGTTGC R-GTAGTTGCAGCTTGAGCCAC	50	(51)
*porA*	F-TCAACTGGACACTTGAAGGTGC R-CCACCATATACGAAGTCAGCACC	52	(51)
*jlpA*	F-GCACACAGGGAATCGACAGC R-AAATGACGCTCCGCCCATTAAC	52	(51)
*virB11*	F-TCTTGTGAGTTGCCTTACCCCTTTT R-CCTGCGTGTCCTGTGTTATTTACCC	55	(9)
*cgtB*	F-TAAGAGCAAGATATGAAGGTG R-GCACATAGAGAACGCTACAA	52	(35)
*wlaN*	F-TGCTGGGTATACAAAGGTTGTG R-ATTTTGGATATGGGTGGGG	54	(42)

1 F – forward; R – reverse

### Detection of virulence genes

Genomic DNA was amplified by PCR to confirm the presence of virulence-associated genes related to adhesion (*flaA, cadF* (*Campylobacter* adhesion to fibronectin), *jlpA* (jejuni lipoprotein A), *porA* (major outer membrane protein) and *pebA* (major cell binding factor)), invasion (*ciaB* (*Campylobacter* invasion antigen B), *pldA* (phospholipase A), *iam* (invasion-associated marker) and *virB11* (type IV secretion system adenosine triphosphatase component)), cytotoxicity (*cdtA, cdtB* and *cdtC* (cytolethal distending toxins A, B and C)), and GBS syndrome (*wlaN* (lipooligosaccharide biosynthesis locus gene N) and *cgtB* (*Campylobacter* galactosyltransferase B)). The PCR reaction mixture of 50 μL volume consisted of 5 μL of 10× PCR buffer, 5 μL of dNTPs (final concentration of 200 μM), 0.5 μL of each primer (final concentration of 0.1 μM), 10 μL of MgCL_2_ (final concentration of 5 mM), 2 μL (2 U) of thermostable Taq polymerase (Thermo Fisher Scientific, Waltham, MA, USA), 5 μL of template DNA (120 ng, as determined with the Nano-Drop Spectrophotometer, Thermo Fisher Scientific, Wilmington, DE, USA) and 22 μL of DNase- and RNase-free deionised water. The amplification protocol included an initial denaturation step at 94°C for 2 min, followed by 35 cycles of denaturation at 94°C for 1 min, annealing at the primer-specific temperature for 1 min and extension at 72°C for 1 min. The annealing temperatures for individual genes are listed in Table 1. Final extension was carried out at 72°C for 5 min. Positive controls, consisting of DNA extracted from *C. jejuni* American Type Culture Collection (ATCC) 33291 and *C. coli* ATCC 43478, as well as a non-template PCR control (PCR-grade water), were included in each PCR run. The PCR products were analysed by gel electrophoresis on a 2% agarose gel stained with ethidium bromide at 5 μg/mL. The sizes of the amplification products were determined using a 100-base-pair (bp) molecular weight marker.

### Sequencing of *flaA*-SVR

The *flaA*-SVR of all isolates was amplified by PCR and subsequently sequenced using the primers listed in Table 1. The PCR conditions were as described previously. The resulting PCR products were visualised by gel electrophoresis, purified using a Clean-Up Kit (A&A Biotechnology), and then sequenced using the Sanger method (Genomed, Warsaw, Poland).

Forward and reverse sequences were assembled and analysed using the Contig Express module in Vector NTI Express (Thermo Fisher Scientific/Life Technologies, Carlsbad, CA, USA), and the sequences were trimmed to a 321-bp region covering the *flaA*-SVR. The alleles were assigned *flaA*-SVR numbers based on the PubMLST database (28). A cluster analysis was performed using default parameters in MEGA X v. 10.1 (31). The maximum likelihood tree based on *flaA*-SVR sequences was visualised using the Interactive Tree of Life (iTOL) platform v. 4 (33). Depositing of the obtained sequences in the GenBank database followed, and the sequences were assigned accession Nos OL314289–OL314318. The genetic diversity of *Campylobacter* isolates from backyard poultry was assessed by calculating Simpson’s diversity index (SDI) with the Comparing Partitions online tool (3).

### Antimicrobial susceptibility testing

The AMR profiles of *Campylobacter* strains were analysed using the minimal inhibitory concentration (MIC) method with the agar dilution technique based on the guidelines of the European Committee on Antimicrobial Susceptibility Testing (EUCAST) (12). The MICs of four antimicrobial agents were determined: erythromycin, a macrolide; ciprofloxacin (CIP), a fluoroquinolone; tetracycline (TE); and gentamicin (CN), an aminoglycoside.

The inocula for each selected colony were prepared in Mueller–Hinton broth (Biomaxima, Lublin, Poland) at a density corresponding to a 0.5 McFarland turbidity standard. They were then diluted 1 : 10 to achieve a final concentration of 10^4^ CFU/mL. Using a Steers multipoint replicator, the inocula were transferred onto pre-prepared Mueller–Hinton agar plates containing serial twofold dilutions of each antimicrobial agent ranging from 0.015 to 64 mg/L for E and CIP, and from 0.03 to 128 mg/L for TE and CN. The plates were incubated in a microaerobic environment for 24 h. The MIC was defined as the lowest concentration of the antimicrobial agent that completely inhibited visible growth of the target organism. *Campylobacter jejuni* ATCC 33560 was used as the control for antimicrobial susceptibility tests. The strains were classified as susceptible or resistant based on the epidemiological cut-off values recommended by EUCAST. The cut-off values for E, CIP and TE were determined according to the EUCAST breakpoints for *Campylobacter*. However, gentamicin breakpoints for *Campylobacter* have not been specified by EUCAST, and therefore the cut-off values for CN were determined based on the breakpoints for Enterobacteriaceae (12). Strains exhibiting resistance to at least one antimicrobial drug in three or more antimicrobial classes were classified as multidrug-resistant (MDR) (37).

### Statistical analysis

Statistical analyses were conducted using Statistica v. 13.3 (TIBCO, Palo Alto, CA, USA). The presence of virulence genes and antimicrobial resistance profiles was assessed using the Kruskal–Wallis test, a non-parametric equivalent to one-way ANOVA, followed by pairwise comparisons using the non-parametric Mann–Whitney U test. A P-value of <0.05 was considered statistically significant.

## Results

### Bacterial strain isolation and identification

A total of 16 *Campylobacte*r spp. isolates were recovered from the 315 cloacal swabs, yielding a prevalence rate of 5.1%. An analysis of host-associated prevalence revealed that *Campylobacter* spp. were detected in 10.0% of ducks (6 out of 60) and 4.7% of ornamental chickens (10 out of 215). None of the cloacal swabs from geese tested positive for *Campylobacter* spp. All strains isolated from *Campylobacter*-positive ducks (6/6; 100.0%) were identified as *C. jejuni*. In ornamental chickens, most isolates were classified as *C. jejuni* (8/10; 80.0%), while *C. coli* was detected in two *Campylobacter*-positive samples (2/10; 20.0%). Notably, both *C. coli* isolates originated from ornamental chickens kept in the same backyard flock (No. 4). A detailed characterisation of individual *Campylobacter* isolates, including their source and molecular data, is provided in Supplementary Table 1.

As regards the location of the studied backyards (Table 2), most swabs were collected in rural environments (265 swabs from four sampling sites), and the remaining samples originated from two semi-urban holdings (50 swabs in total). *Campylobacter* spp. isolates were obtained from both rural areas (seven positive samples) and semi-urban ones (nine positive samples). The prevalence of *Campylobacter* reached 18.0% in semi-urban environments and 2.6% in rural environments. The chi-squared test indicated that the difference between these groups approached statistical significance (χ^2^ = 3.64, P-value = 0.056). However, this apparent difference should be interpreted with caution, given the small number of holdings and the fact that most positive samples originated from a single semi-urban site. Notably, *Campylobacter* was detected in all backyards where birds were kept together with other animals (such as dogs or cats), whereas the backyard without such cohabitation remained *Campylobacter* free.

**Table 2.** Characteristics of backyard Polish poultry holdings and sampled birds

Backyard No.	Location	Number and species of birds sampled	Breeds samples	Other animals kept			Number of positive samples (%)
				Dogs and cats	Others	Total	
1	Rural area	35 ducks 10 geese	Polish Pekin duck Toulouse goose	3 dogs	8 goats 4 horses	15	3/45 (6.7%)
2	Rural area	15 ducks 30 geese 25 ornamental chickens	Khaki Campbell duck Polish White goose Satsumadori, Yamato, Ayam Cemani	6 dogs 4 cats	0	10	3/70 (4.3%)
5	Rural area	94 ornamental chickens	Araucana, Leghorn, Shamo, Denizli, Satsumadori, Yamato, Ko-Shamo, Ayam Cemani, Polish Bantam	0	0	0	0/94 (0.0%)
6	Rural area	56 ornamental chickens	Silkie, Shamo, Chabo, Onagadori, Ayam Cemani, Bantam Cochin	2 dogs 2 cats	6 goats	10	1/56 (1.8%)
3	Semiurban	10 ducks	Polish Pekin duck	2 dogs 12 cats	2 cows 6 pigs	22	1/10 (10.0%)
4	Semiurban	40 ornamental chickens	Onagadori, Ko-Shamo, Tomaru, Chabo, Totenko, Ohiki, Minohiki, Silkie	2 dogs 7 cats	0	9	8/40 (20.0%)

As regards flock distribution, the highest proportion of *Campylobacter* isolates originated from backyards No. 3 (10.0%) and No. 4 (20.0%), both located in semi-urban areas. The proportions of isolates from backyards No. 2 and No. 6 were significantly lower, accounting for only 4.3% and 1.8%, respectively (P-value < 0.05).

### Detection of virulence genes

The 14 *C. jejuni* and 2 *C. coli* strains which had been isolated were tested for the presence of the 14 virulence-associated genes stated previously. The *flaA* gene was universally present in all isolates (100.0%). The *cadF* gene was the second most prevalent adhesion marker; being detected in 62.5% of all isolates, in 64.3% (9/14) of those of *C. jejuni* and in 50% (1/2) of *C. coli*. This gene was more frequently identified in strains from ornamental chickens (80.0%, 8/10) than ducks (33.3%, 2/6). The other adhesion-associated genes were considerably rarer, *porA* being detected in 12.5%, *pebA* also in 12.5% and *jlpa* in 6.3% of total isolates. These genes were identified exclusively in *C. jejuni* isolates, predominantly those obtained from ducks.

The most common invasion gene was *ciaB*, and it was found in 75.0% (12/16) of all isolates and in 85.7% (12/14) of *C. jejuni* isolates. This gene was more prevalent in duck isolates (83.3%, 5/6) than in ornamental chicken isolates (70.0%, 7/10). The *pldA* gene was detected only in *C. jejuni* strains (35.7%, 5/14), all derived from ornamental chickens. The *iam* gene was rare and detected in only one *C. coli* isolate from an ornamental chicken. The *virB11* gene, which also contributes to bacterial invasiveness *via* the type IV secretion system, was found in 14.3% (2/14) of *C. jejuni* isolates, all of which originated from ducks. The *virB11* gene was not detected in *C. coli* isolates.

Among the genes in the cytotoxin-producing *cdt* cluster, *cdtC* was the most frequently identified, present in 62.5% (10/16) of all isolates. It was detected in 71.4% (10/14) of those of *C. jejuni*, and absent from *C. coli*, as were the other cluster genes. The *cdtB* gene was found in 50.0% (7/14) of *C. jejuni* isolates, and *cdtA* was the least common, detected in only 14.3% (2/14). Notably, duck-derived isolates exhibited higher frequencies of both *cdtB* (83.3%, 5/6) and *cdtC* (83.3%, 5/6), compared to isolates from ornamental chickens (20.0% for *cdtB* (2/10) and 50% for *cdtC* (5/10)).

Genes encoding the biosynthesis of sialylated lipooligosaccharides (LOSsial), compounds that are implicated in the development of GBS, were rarely detected. The *cgtB* gene was identified in two duck-derived *C. jejuni* isolates (12.5% of the total for both *Campylobacter* species), while *wlaN* was not detected in any of the tested strains.

Clusters were also sought besides single genes. In the examined isolates, *cdtB*-*cdtC* was the most common combination of cytotoxicity-related genes and was observed in 37.5% (6/16) of all strains. A complete *cdtA-cdtB-cdtC* cluster was identified in only one *C. jejuni* isolate (6.25%). The *ciaB-pldA* pattern occurred in four *C. jejuni* isolates, all derived from ornamental chickens (Table 3).

**Table 3.** Gene patterns and profiles of *Campylobacter jejuni* isolates from backyard-reared Polish ducks and ornamental chickens

Gene profile	Gene pattern	Number of isolates (%)
**adhesion**	*pebA-porA-jlpA*	1/14 (7.1)
**invasion**	*ciaB-virB11*	2/14 (14.3)
	*ciaB-pldA*	4/14 (28.6)
**cytotoxicity**	*cdtA-cdtB-cdtC*	1/14 (7.1)
	*cdtA-cdtB*	1/14 (7.1)
	*cdtA-cdtC*	2/14 (14.3)
**GBS**	*cgtB*	2/14 (14.3)

1 GBS – Guillain-Barré syndrome

### *FlaA*-SVR sequencing

**Fig. 1.**
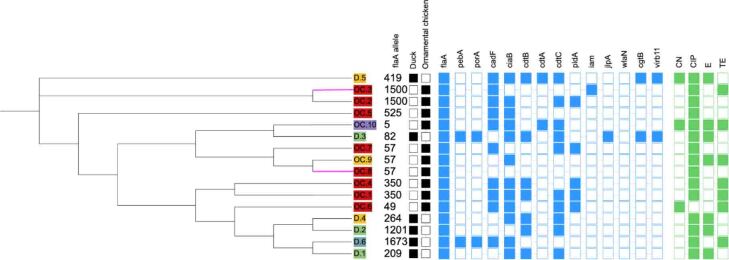
Maximum-likelihood phylogenetic tree of *Campylobacter* isolates from backyard poultry in Poland. Green – Backyard No. 1; yellow – backyard No. 2; blue – backyard No. 3; red – backyard No. 4; purple – backyard No. 6. Solid black square – species of origin; hollow white square – not species of origin; solid blue square–virulence gene detected; hollow blue square – virulence gene not detected; solid green square – resistance exhibited; hollow green square – resistance not exhibited. Branches highlighted in pink on the tree represent *C. coli* isolates. CN – gentamicin; CIP – ciprofloxacin; E – erythromycin; TE – tetracycline

The analysis of *flaA*-SVR sequences in 16 *Campylobacter* isolates revealed a total of 12 different alleles (Fig. 1). Allele 57 was most prevalent, and it was identified in three isolates (18.8%), all of which originated from ornamental chickens. These strains accounted for 30.0% (3/10) of all isolates derived from ornamental chickens. Nine alleles were identified as unique, each occurring in only one isolate.

Duck isolates were most diverse (n = 6), and each isolate had a unique *flaA*-SVR allele (alleles 419, 82, 264, 1201, 1673 and 209). In these isolates, the SDI reached 1.000, indicating complete allelic variation. Six distinct alleles (alleles 1500, 525, 5, 57, 350 and 49) were identified in the isolates from ornamental chickens (n = 10), and three alleles (1500, 57 and 350) were found in multiple isolates. These results yielded an SDI of 0.911 (95% CI: 0.832–0.970), which indicates that allelic diversity was only slightly lower than in duck isolates.

### Antimicrobial resistance

The studied strains were most frequently resistant to ciprofloxacin (15/16 of resistant isolates), next most frequently to tetracycline (7/16) and erythromycin (7/16) and least frequently resistant to gentamicin (3/16) (Table 4). All isolates were resistant to at least one antimicrobial agent. Seven distinct resistance profiles were identified, the most common being CIP-TE (37.5%), E-CIP (43.6%), and CIP alone (25.0%). The other, less frequently observed profiles were E-CIP-TE (12.5%), E-CIP-CN (6.3%), TE-CN (12.5%) and E-CIP-TE-CN (6.3%). Notably, all six duck isolates were resistant to at least two antimicrobials. In contrast, four of the ten isolates from ornamental chickens were resistant to only one tested antimicrobial agent.

**Table 4.** Distribution of MICs for *Campylobacter* strains (n = 16) isolated from Polish backyard poultry

Antimicrobial agent	Number of isolates with the specific MIC (mg/L)														Resistance (%)
	0.015	0.03	0.06	0.12	0.25	0.5	1	2	4	8	16	32	64	128	
TE				3	3	1	2		1	1	1	2	1	1	43.6
E				2	2	2	3		2	2	1	2			43.6
CN				2	2	3	2	4				1	1	1	18.8
CIP						1				1	1	4	5	4	93.8

1 Shaded areas indicate the susceptibility range of each antibiotic tested. MIC – minimum inhibitory concentration; TE – tetracycline; E – erythromycin; CN – gentamicin; CIP – ciprofloxacin

In analysis, 3 of the 16 *Campylobacter* strains (18.8%) were classified as MDR according to the criteria proposed by Magiorakos *et al*. (37). The most frequent MDR patterns included resistance to macrolides, fluoroquinolones and tetracyclines (1/16) and macrolides, fluoroquinolones and aminoglycosides (1/16), each accounting for 6.3% of the isolates. One isolate from an ornamental chicken (6.3%) exhibited resistance to all four tested antimicrobial classes.

At the flock level, all three isolates (100%) from backyard No. 1 were characterised by the E-CIP resistance profile. Similarly, all three isolates from backyard No. 2 were resistant to ciprofloxacin and erythromycin, where one isolate from an ornamental chicken had an extended E-CIP-TE resistance profile, and one duck isolate had an extended E-CIP-CN resistance profile. A single strain with a CIP-TE resistance pattern was isolated from backyard No. 3. Seven of the eight strains isolated from backyard No. 4 were resistant to ciprofloxacin. Of these, four isolates were resistant only to ciprofloxacin, while three isolates exhibited the combined CIP-TE resistance pattern. A single strain resistant to all tested antimicrobials was isolated from backyard No. 6 (Fig. 1).

## Discussion

*Campylobacter* is now widely recognised as a leading pathogen borne in food potentially contaminated in intensive poultry production systems, where flock density, standardised management and centralised slaughter chains are primary contributors to contamination risk (21). The existing literature has predominantly concentrated on its occurrence in these poultry production systems, while the role of small-scale or backyard poultry rearing in *Campylobacter* spread remains comparatively underexplored, despite its increasing popularity and how closely birds kept there interact with humans and other domestic species.

The overall prevalence of *Campylobacter* in domestic animals varies widely. It has been noted to reach 80.0% in pigs and to range from 6.8% to 16.1% in small ruminants, 4.6% to 50.2% in broilers and 1.4% to 37.0% in cats and dogs (13, 34, 40, 52, 62). In other animals, the prevalence of *Campylobacter* has been determined at 11.7%, and most cases have been reported in terrestrial game mammals (13.0%) and birds (9.3%) (13). The primary aim of this study was to evaluate the prevalence and distribution of *Campylobacter* in domestic poultry raised in small backyard holdings in diverse environments. The study revealed that the prevalence of *Campylobacter* spp. was low in all analysed samples (5.1%). This value is considerably lower than those noted in previous studies investigating backyard or small-scale poultry rearing. For example, Anderson *et al*. (1) reported a prevalence of up to 86.0% in backyard poultry flocks in New Zealand, while Parzygnat *et al*. (47) documented a 21.9% prevalence in similar flocks in the USA. Higher prevalence rates of *Campylobacter* spp. have also been reported in commercial broiler production systems. For instance, a United Kingdom-wide survey found that 79.2% of broiler flocks tested positive for *Campylobacter* at slaughter, and the infection risk increased with age and in the summer months (32). Similarly, studies conducted in the Netherlands reported a 30.0% prevalence in broiler flocks at slaughter age, highlighting that bird age and environmental conditions were significant risk contributors (46). Backyard poultry are often kept without adherence to formal sanitation or biosecurity protocols, which might suggest that the low prevalence observed in the present study could be unexpected. However, factors such as lower flock density and reduced turnover may contribute to less consistent transmission dynamics (54). It is important to note that the variability in *Campylobacter* prevalence reported across different studies is likely multifactorial. These variations can stem from differences in methodological approaches, including diverse sampling techniques, geographic and environmental variables, seasonal influences, and the specific hygiene and handling practices in different countries.

Campylobacter was detected in domestic poultry from both rural and semi-urban environments, with flock-level prevalence reaching 2.6% and 18.0%, respectively. This observation suggests that semi-urban environments may represent conditions more conducive to *Campylobacter* occurrence in backyard poultry, potentially increasing the risk of transmission to humans. However, these findings should be interpreted with caution. Although the difference in prevalence between rural and semi-urban environments did not reach statistical significance, the observed trend may indicate increased exposure of poultry to environmental sources of contamination in semi-urban settings. Such conditions may include higher animal density, closer proximity between households, and increased interaction with other domestic or synanthropic animals. Further studies involving larger sample sizes are required to confirm this association and to better understand the underlying drivers of *Campylobacter* transmission in different backyard environments.

The highest detection rate of *Campylobacter* carriers was found in ducks (10.0%), and the next highest in ornamental chickens (4.7%). This study’s anatine prevalence of *Campylobacter* spp. being highest is consistent with the observations made in other regions and suggests that waterfowl are an important reservoir of this pathogen (60). In a study conducted in South Korea, the prevalence of *Campylobacter* spp. was substantially higher in duck isolates than in chicken isolates (77.5% *vs* 32.0%) (6). Ornamental chickens showed a comparatively low prevalence, although not the lowest among the studied species. Although very few studies have focused specifically on ornamental or hobbyist poultry, previous research suggests that lower flock density and restricted outdoor access in such settings may contribute to reduced environmental exposure and pathogen transmission (48).

The non-detection of *Campylobacter* spp. in any sampled geese stands in contrast to previous studies, where considerably higher prevalence rates were reported in this avian species. For example, a study conducted at the Oxford University Farm in the UK identified 166 *C. jejuni* isolates from 331 faecal samples collected from geese, corresponding to a prevalence rate of 50.2% (7). Similarly, Wysok *et al*. (61) detected *Campylobacter* spp. in 83.3% of caecal content samples from domestic geese in Poland. These discrepancies may reflect differences in sampling strategies or diagnostic sensitivity, or seasonal and ecological factors that affect bacterial shedding.

At the flock level, *Campylobacter* was detected in five out of the six examined backyard flocks. The prevalence within individual flocks ranged from 1.8% to 20.0%, indicating substantial variability in flock colonisation rates. The highest prevalence was noted in backyard No. 4, where 8 out of 40 samples tested positive. This flock consisted of ornamental chickens that were raised in proximity to dogs and cats, suggesting potential interspecies transmission or shared sources of environmental contamination. In contrast, *Campylobacter* was not detected in poultry in backyard No. 5, where other animal species were not kept. These observations may indicate an association between the presence of additional animals and the occurrence of *Campylobacter* in backyard flocks; however, no direct conclusions regarding interspecies transmission can be drawn from the present study. Further investigation is therefore required to clarify the role of multi-species environments in pathogen dynamics. Such a possibility has been previously suggested, with interspecies transmission between birds and mammals in non-commercial settings proposed as a potential factor contributing to zoonotic risk (45). Consequently, even in the absence of intensive production pressures, pathogen circulation may be influenced by shared spaces, feed, and water systems (17).

*Campylobacter jejuni* isolates from ducks and ornamental chickens were characterised by high genetic diversity as well as diverse virulence profiles. In these hosts, a broader distribution of *cadF* and other adhesion genes (especially prevalent in ornamental chickens) and the presence of multiple combinations of the *ciaB, pldA* and *virB11* invasion-associated genes point to the heterogeneous nature of the *Campylobacter* populations circulating in more complex ecological or management contexts (44). The birds kept in those contexts, particularly ducks, may be exposed to environmental sources such as surface water or free-ranging flocks, which are known to harbour diverse *Campylobacter* genotypes and virulence patterns (44, 60).

Moreover, the host-specific distribution of the *cdtABC* cluster further highlights the differential pathogenic potential of *Campylobacter* strains across bird species. Although *cdtC* was more widely distributed in both ducks and ornamental chickens, the entire *cdtABC* cytotoxin gene cluster was more commonly detected in duck-derived isolates, suggesting a higher cytotoxic potential in this group. Since cytolethal distending toxin production is associated with DNA damage and host cell-cycle arrest, the higher prevalence of this cluster in ornamental chickens may indicate increased virulence potential, at least at the genomic level (27). Despite the non-detection of the entirety of the *cdtABC* operon in any of the isolated strains, partial gene presence may still support cytotoxic activity, as shown in other avian isolates (26).

The genes associated with the biosynthesis of LOSsial, namely *wlaN* and *cgtB*, were infrequently detected in the examined isolates. These genes are of particular interest because they encode structural mimics of host gangliosides and in this way may trigger post-infectious neuropathies such as GBS (18). The absence of the *wlaN* gene and the low prevalence of *cgtB* (12.5%), which was detected only in duck isolates, suggest that although the zoonotic potential of the isolated avian *Campylobacter* strains cannot be ruled out, their capacity to induce severe sequelae such as GBS appears limited. The overall prevalence of virulence genes was markedly lower in *C. coli* isolates than in *C. jejuni* ones. Except for one strain carrying the *ciaB* gene and another harbouring the *iam* gene, no additional virulence determinants were detected in *C. coli* samples. This observation aligns with earlier findings which revealed a more limited virulence repertoire in *C. coli*, suggesting that this pathogen and *C. jejuni* could differ in their host ranges, ecological niches or pathogenesis (45).

The genetic diversity of *Campylobacter* isolates was evaluated through the sequencing of the *flaA*-SVR, which revealed considerable differences between those from one avian host and those from the other. Each of the six isolates derived from ducks possessed a distinct *flaA*-SVR allele (419, 82, 264, 1201, 1673 or 209), which resulted in maximal diversity (SDI = 1.000). In contrast, the ten isolates from ornamental chickens were clustered into six alleles (1500, 525, 5, 57, 350 or 49), yielding a lower but still high SDI of 0.911 (95% CI: 0.832–0.970). Notably, allele 525, which was identified in a single isolate from an ornamental chicken, and duck-derived alleles 1201, 1673 and 209 were absent from the PubMLST database, indicating that potentially novel genotypes may circulate in backyard settings. However, the chicken-derived alleles 57, 350 and 49 have already been documented in poultry and environmental sources, suggesting that these strains may have broader ecological and interspecies distributions (10). The slightly higher allelic heterogeneity in ducks than in ornamental chickens points to distinct ecological reservoirs and evolutionary dynamics within backyard poultry flocks. This observation is consistent with the findings of Dingle *et al*. (21), who demonstrated that genetically diverse *Campylobacter jejuni* populations, as defined by sequence-based typing methods, are widely distributed across different hosts and environmental sources, and that certain genotypes can be shared between animal reservoirs and humans. Their work highlighted that high allelic diversity reflects frequent genetic recombination and adaptation to multiple ecological niches, which may facilitate the persistence and transmission of strains with zoonotic potential. In this context, the coexistence of multiple *flaA* alleles within backyard poultry systems may indicate the circulation of genetically diverse strains capable of crossing host boundaries and contributing to human infection.

The detection of antimicrobial-resistant *Campylobacter* isolates in this study is consistent with the globally reported increase in AMR in foodborne pathogens (13). Resistance to tetracycline and gentamicin is of particular concern. These drugs are critical therapeutic alternatives, especially in patients with systemic campylobacteriosis (15). In the present study, 93.8% of *Campylobacter* isolates were resistant to ciprofloxacin, 43.8% to tetracycline, 43.8% to erythromycin and 18.8% to gentamicin. The observed resistance rates were higher than those noted by other authors (2), but consistent with the findings reported by Mousavinafchi *et al*. (41), Iqbal *et al*. (25) and Popa *et al*. (50), indicating that resistance to these antimicrobials is geographically variable but remains a consistent challenge. Given that AMR in *Campylobacter* is increasingly recognised as a global priority (59), these results reinforce the need to monitor and mitigate resistance in avian populations kept outside traditional production systems.

The presence of MDR *Campylobacter* spp. strains in this study raises substantial public health concerns. Specifically, 18.8% of the isolated *Campylobacter* spp. strains were classified as MDR, most commonly exhibiting resistance to macrolides, fluoroquinolones and tetracyclines. Combined resistance to tetracyclines and fluoroquinolones was also reported by Popa *et al*. in 2025 (50). Moreover, the prevalence of MDR strains was similar to that noted in a Canadian study of broiler chickens (11). In that experiment, 18.7% of *Campylobacter* isolates exhibited resistance to multiple antimicrobials. The relatively high prevalence of MDR *Campylobacte*r isolates in the current study indicates that therapeutic options may be limited during campylobacteriosis outbreaks caused by these strains.

Several limitations inherent in the study design should be acknowledged. Firstly, the research was geographically limited to the north-eastern region of Poland, where all the backyard flocks were located. The findings may not accurately represent the situation in other parts of the country, as regional differences in *Campylobacter* prevalence and AMR patterns may exist. Also, there was a temporal limitation, because the samples were collected during the spring season, which could have influenced the observed prevalence, given the known seasonal variation in *Campylobacter* occurrence. Although sampling was designed to reflect a cross-section of the poultry species, management practices and urban-rural settings typical of backyard flock rearing, accessibility and owner willingness were also factors in site selection; the sample may therefore be only imperfectly representative. Moreover, the limited number of analysed flocks may constrain the generalisability of the present findings. Consequently, although the results provide valuable insights into the prevalence, genetic diversity and AMR of *Campylobacter* spp. in small poultry flocks in Poland, they should be interpreted cautiously.

## Conclusion

The results of this study highlight the epidemiological relevance of backyard poultry, particularly in environments where birds are in close proximity to humans. Although the overall prevalence of *Campylobacter* spp. was lower than that typically reported in commercial poultry systems, the detection of genetically diverse and potentially virulent strains emphasises the importance of continued surveillance in non-commercial settings. The co-occurrence of positivity in poultry with the keeping of other animal species where *Campylobacter* was detected warrants further investigation into the potential role of shared animal environments in pathogen dynamics and transmission. These results reinforce the need for targeted biosecurity measures even in small or hobbyist poultry rearing. Overall, this study advocates for the inclusion of backyard poultry and the associated animal reservoirs in national surveillance programmes, the implementation of tailored hygiene strategies in smallholder settings and broader recognition of hobbyist flocks as potential contributors to the zoonotic transmission of virulent and resistant *Campylobacter* strains.
